# Soybean Lectin Cross-Links Membranes by Binding Sulfatide in a Curvature-Dependent Manner

**DOI:** 10.1021/acs.jafc.5c04336

**Published:** 2025-05-24

**Authors:** Ayoyinka O. Okedigba, Emery L. Ng, Mawuli Deegbey, M. Luciana Rosso, William Ngo, Ruoshi Xiao, Haibo Huang, Bo Zhang, Valerie Vaissier Welborn, Daniel G. S. Capelluto

**Affiliations:** † Protein Signaling Domains Laboratory, Department of Biological Sciences, Fralin Life Sciences Institute, and Center for Soft Matter and Biological Physics, 1757 Virginia Tech, Blacksburg, Virginia 24061, United States; ‡ Department of Chemistry, Virginia Tech, Blacksburg, Virginia 24061, United States; § Facility for Advanced Imaging and Microscopy, Fralin Life Sciences Institute, Virginia Tech, Blacksburg, Virginia 24061, United States; ∥ Macromolecules Innovation Institute, Virginia Tech, Blacksburg, Virginia 24061, United States; ⊥ School of Plant and Environmental Sciences, Virginia Tech, Blacksburg, Virginia 24061, United States; # Department of Food Science and Technology, Virginia Tech, Blacksburg, Virginia 24061, United States

**Keywords:** lectin, soybean, meal, *N*-acetyl-d-galactosamine, sulfatide, membrane curvature

## Abstract

Soybean (*Glycine max*) is a key source of plant-based protein, yet its nutritional value is impacted by antinutritional factors, including lectins. Whereas soybean lectin is known to bind *N*-acetyl-d-galactosamine (GalNAc), its lipid interactions remain unexplored. Using a novel purification method, we isolated lectin from soybean meals and characterized its interactions with GalNAc and the glycosphingolipid sulfatide. Isothermal titration calorimetry revealed micromolar affinity for GalNAc, whereas most GalNAc derivatives displayed weak or no binding. Lectin exhibited high-affinity binding to sulfatide in a membrane curvature-dependent manner. Binding of lectin to sulfatide promoted cross-linking of sulfatide-containing vesicles. Whereas sulfatide interaction was independent of GalNAc binding, suggesting distinct binding sites, vesicle cross-linking was inhibited by the sugar. Molecular dynamics simulations identified a consensus sulfatide-binding site in lectin. These findings highlight the dual ligand-binding properties of soybean lectin and may provide strategies to mitigate its antinutritional effects and improve soybean meal processing.

## Graphical abstract

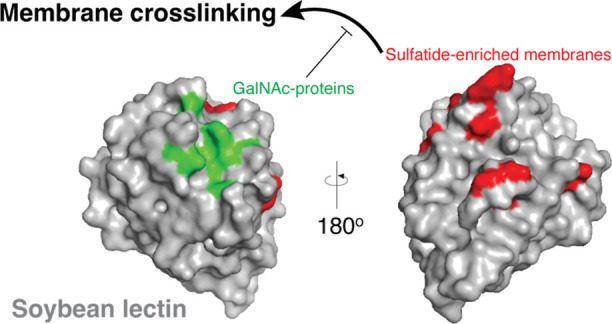

## 1 Introduction

Soybean [*Glycine max* (L.) Merr.] stands as one of the foremost sources of plant-based protein and oil worldwide, contributing to both human and animal nutrition. Beyond its value as a food crop, soybean also serves as a critical source of metabolizing energy, playing major roles in livestock, poultry, and aquaculture production systems globally. Rich in essential amino acids, polyunsaturated fatty acids, and bioactive compounds, soybeans are valued for their high nutritional profile. Recent nutritional studies underscore the extensive health benefits of soybean seed consumption, particularly in preventing and managing chronic diseases. These benefits include supporting cardiovascular health through blood pressure regulation, improving lipid metabolism, and reducing cholesterol levels.^1^ In addition, soybean is known for its anticarcinogenic, antibacterial, and anti-inflammatory properties, attributed largely to its isoflavones and peptides.^1,2^ Indeed, soy-derived bioactive peptides may positively impact gut microbiota, enhancing gut health and immune function,^3^ as well as memory.^4^

Despite its advantages, soybean meal contains antinutritional factors that limit its bioavailability and effectiveness in animal diets. Among these factors in legumes are trypsin inhibitors (TIs) and lectins. Lectins, also known as agglutinins, are glycoproteins widely found in microorganisms, plants, and animals, capable of cross-linking animal blood cells.^5^ Soybean lectin adversely impacts animal intestinal health by affecting intestinal structure, barrier function, mucosal immunity, and gut microbiota balance.^6^ Canonical soybean lectins are organized as homotetramers, each composed of 30 kDa subunits.^7^ Each lectin subunit contains a single carbohydrate-binding domain (CBD) that exhibits specificity for *N*-acetyl-d-galactosamine (GalNAc)^8,9^ and is covalently modified by an N-linked high-mannose glycan chain, Man_9_, which may influence lectin stability and binding affinity.^10^ Structurally, each monomer in plant lectins adopts a classic jelly roll fold, composed of 13 β-strands organized into two opposite β-sheets.^11^ The first β-sheet, consisting of seven curved β-strands, harbors the CBD. The activity of this binding site relies on the coordination of Mn^2+^ and Ca^2+^, which are essential for stabilizing the interactions with carbohydrate ligands. The second β-sheet, containing six β-strands, plays a structural role by mediating monomer–monomer interactions, facilitating the assembly of the tetramer through hydrogen bonds and van der Waals forces.^12^

Animal lectins serve as a direct defense mechanism against pathogens and play a role in immunity regulation. A specific group of animal lectins, known as galectins, promotes glycoprotein receptor internalization through endocytosis.^13^ An endocytosis hypothesis suggests that carbohydrate-dependent oligomerization of galectins is a critical step for their association with cell membranes. The narrow membrane bending induced by these activated galectins depends on their interaction with cell surface sphingolipids,^14,15^ leading to the formation of endocytic pits that eventually pinch off to initiate clathrin-independent endocytosis of glycoprotein receptors.^16^ Glycosphingolipids serve as receptors for lectins, and their distribution in specialized membrane domains, known as lipid rafts, is essential for cell–cell communication and adhesion.^17^ Among glycosphingolipids, sulfatide plays a key role in regulating adhesive receptors, innate immune receptors, and coagulation factors at the cell surface^18^ by facilitating electrostatic interactions with proteins through its negatively charged headgroup.^19^ Mammalian galectin-8 has been shown to interact with glycosphingolipids, including sulfatide, with high affinity,^20^ whereas galectin-4 accumulates in hippocampal and cortical neurons in a microtubule- and sulfatide-dependent manner.^21^ Galectin-4 binding to sulfatide-containing membranes has been proposed to stabilize glycolipid-rich domains in association with specific glycoproteins.^22^ Early studies indicate that soybean lectin primarily binds to glycolipids in pig lymphocyte plasma membranes, with trihexosyl ceramide and globoside serving as the major binders, whereas ganglioside GM2 exhibits a lower binding affinity.^23^ However, given the low sequence homology between soybean lectins and their mammalian counterparts, it remains unclear whether they can also recognize sulfatide for host membrane targeting.

Here, we show a simple three-step protocol capable of isolating lectin, along with TIs, including Kunitz TI (KTI) and Birk and Bowman TI (BBTI), from soybean meal. The purified lectin was identified as a homotetramer that exhibited a higher affinity for GalNAc than other known GalNAc analogs reported to bind mammalian lectins. Also, soybean lectin showed nanomolar affinity for sulfatide in a membrane curvature-dependent manner. GalNAc and sulfatide did not influence each other’s binding, suggesting that they interact with distinct regions on lectin. Molecular dynamics simulations (MDS) identified the presence of a sulfatide-binding site located opposite the CBD. The association of lectin with sulfatide induced local conformational changes in the protein, leading to the cross-linking of sulfatide-containing vesicles, a process that was downregulated by GalNAc.

## 2 Material and Methods

### 2.1 Chemicals

Lectin, cholesterol, KTI, and BBTI standards were purchased from Sigma-Millipore. Dioleoylphosphatidylcholine (DOPC), dioleoylphosphatidyl ethanolamine (DOPE), 1,2-dioleoyl-*sn*-glycero-3-phosphoethanolamine-*N*-(carboxyfluorescein) (DOPE-F), and brain sulfatide were obtained from Avanti Research. GalNAc was purchased from Sigma-Millipore. 2-Nitrophenyl-β-d-galactopyranoside (NPGP) , 4-nitrophenyl-α-d-mannopyranoside (NPMP), and d-(+)-galactose were acquired from ThermoScientific. 6′-Sialyl lactose (6-SL) was purchased from Cayman Chemicals. Methyl-*N*-acetyl-2-deoxy-α-d-galactosamine (MGalNAc) was purchased and lacto-N-neotetraose (L-NT) was obtained from Toronto Research Chemicals. Octyl-α-d-mannopyranoside (OMP) and 2-chloro-4-nitrophenyl-α-d-mannopyranoside (CNMP) were purchased from Biosynth. All other reagents were of analytical grade.

### 2.2 Plant Material

The Glenn soybean cultivar, developed at Virginia Tech, serves as a reference variety. Additional soybean breeding lines with varying TI activities were generated by crossing PI 547656 × Glenn, PI 547656 is a low-TI cultivar obtained from the USDA Soybean Germplasm Collection in Urbana, IL.

### 2.3 Meal Processing

Soybean meal was produced as described previously.^24^

### 2.4 Soybean Lectin and TIs Purification

Lectin was extracted from pulverized soybean meal using 0.1 M sodium acetate buffer (pH 4.6) by incubating the mixture for 2 h at 25 °C. After extraction, the mixture was centrifuged at 1321*g* for 30 min at 4 °C. The resulting supernatant was carefully decanted, microfiltered using 0.45 μm syringe filters, and incubated with GalNAc beads (Millipore Sigma) for 2 h. Following incubation, the TI-enriched nonbound fraction was concentrated to 2 mL using a 3 kDa concentrator (Sigma-Millipore) at 1321*g* at 4 °C. The beads were thoroughly washed with 0.1 M sodium acetate buffer (pH 4.6), and lectin was eluted twice using 0.2 M galactose, with each elution performed after a 30 min incubation. The eluted sample was then concentrated using a 3 kDa concentrator (Sigma-Millipore). For further purification, the concentrated sample was processed using an FPLC AKTA PURE system equipped with a Superdex 200 gel filtration column pre-equilibrated with 20 mM Tris–HCl and 500 mM NaCl (pH 7). Fractions corresponding to highly purified lectins were pooled and concentrated. The sample was then buffer exchanged to either 50 mM HEPES, 140 mM NaCl (pH 7.4) for liposome co-sedimentation, surface plasmon resonance (SPR), and circular dichroism (CD) assays or 50 mM HEPES, 20 mM CaCl_2_ (pH 7) for ITC experiments. Separately, the TI-enriched nonbound fraction was loaded onto a Superdex-30 gel filtration column to isolate KTI and BBTI, using 20 mM Tris–HCl and 500 mM NaCl (pH 7). Protein concentration was determined by measuring absorbance at 280 nm using a NanoDrop One instrument (Thermo Fisher Scientific) and estimated through SDS–PAGE analysis, comparing protein band intensities to a glutathione S-transferase (GST) reference.

### 2.5 Mass Spectrometry

Lectin was identified using mass spectrometry analysis at the Mass Spectrometry Core Facility at Virginia Tech. Samples were prepared in 10 mM Tris–HCl (pH 8.5) and digested with 500 ng/μL trypsin at 37 °C for 10 h. The resulting peptides were analyzed on an Orbitrap Fusion Lumos Tribid mass spectrometer (Thermo Fisher Scientific). Data processing was carried out using Proteome Discoverer 2.5 (Thermo Fisher Scientific), integrating search results from Mascot 2.7 and Sequest HT (Matrix Science) into a consolidated data set for protein identification.

### 2.6 Native Gel Electrophoresis

Purified lectin (4 μM) in a buffer containing 50 mM HEPES (pH 7.4) and 140 mM NaCl was pre-equilibrated at room temperature for 15 min and then incubated with or without 20 mM GalNAc for 1 h at room temperature. The samples were then mixed with native gel sample buffer and resolved on a 10% native gel.

### 2.7 Liposome Co-Sedimentation Assay

Lipids (1.6 mg total) in organic solvent were mixed in a glass vial at the following molar ratios: 40% sulfatide, 20% DOPC, 20% DOPE, and 20% cholesterol. The lipid mixture was briefly vortexed and sonicated, then dried under a stream of nitrogen gas. The lipid mixture was subsequently placed in a desiccator under vacuum conditions for 2 h to ensure complete drying. Following this, 0.8 mL of prewarmed 50 mM HEPES and 140 mM NaCl (pH 7.4) buffer was added to the dried lipids (∼2 mg/mL). The lipid mixture was incubated in a 67 °C water bath to hydrate the lipids. The hydrated lipid suspension underwent six freeze–thaw cycles, alternating between 1 min incubation in liquid nitrogen and 3 min in a 67 °C water bath, to promote multilamellar vesicle formation. To obtain unilamellar vesicles, lipid suspensions were extruded through a polycarbonate filter with pore sizes of 0.4, 0.2, 0.1, or 0.05 μm, using a mini extruder (Avanti Research).

For liposome cosedimentation assays, proteins (20 μg) and liposomes (2 mg/mL) were incubated at room temperature for 60 min. The mixtures were centrifuged at 63,500 rpm for 60 min at 20 °C using a TLA 100 rotor (Beckman Coulter) to separate the liposome-bound fraction (pellet) from the liposome-free fraction (supernatant). Equal proportions of supernatant and pellet fractions were subjected to SDS–PAGE. After electrophoresis, the gels were stained with Coomassie blue, and the intensities of the lectin bands were quantified by Image Lab software (Bio-Rad).

### 2.8 Dynamic Light Scattering

DLS experiments were performed at 25 °C using a Malvern Zetasizer Nano-ZS instrument. The studies were carried out with liposomes at a concentration of 2 mg/mL in a buffer containing 50 mM HEPES and 140 mM NaCl (pH 7.4). Each run was recorded for 120 s, and three runs were averaged after a 2 min equilibration period.

### 2.9 Isothermal Titration Calorimetry

Isothermal titration calorimetry (ITC) measurements were performed in a buffer containing 50 mM HEPES and 20 mM CaCl_2_ (pH 7). GalNAc and its analogs (2.5–5 mM) were titrated into 300 μL of 20 μM lectin with a 0.4 μL first injection and 18 subsequent 2 μL injections using a MicroCal PEAQ system (Malvern) equilibrated at 25 °C. For the competition assay, 20 μM soybean lectin was preincubated with ∼300 μM liposomes (0.4 μm) composed of either 10% sulfatide/90% DOPC or 100% DOPC and placed in the ITC sample cell, whereas 2.5 mM GalNAc was loaded in the syringe. Each experiment was carried out in duplicate. In all cases, data were best fitted using a one-set site model with the MicroCal PEAQ-ITC analysis software.

### 2.10 Surface Plasmon Resonance

SPR data were collected using a BIAcore X100 instrument equipped with an L1 sensor chip (Cytiva) coated with 0.4 μm liposomes at room temperature. Liposomes contained 10% sulfatide and 90% DOPC, whereas liposomes without sulfatide were composed of 100% DOPC. All experiments were performed using a buffer consisting of 50 mM HEPES and 140 mM NaCl (pH 7.4). Pretreatment of the sensor chip was performed with 40 mM octyl β-d-glucopyranoside. Liposomes were immobilized onto the L1 sensor chip at a flow rate of 30 μL/min, achieving a typical loading of ∼5500 response units per sensor chip channel. Lectin was used as the analyte across the specified concentration range. For the competition assays, soybean lectin was prepared at various concentrations, ranging from 0.05 to 10 μM, each containing 1 mM GalNAc. Data were best fitted using a two-state reaction model, and the apparent *K*_D_ values were estimated using BIAevaluation software, version 2.0 (Cytiva).

### 2.11 Circular Dichroism

Near-UV CD spectra were recorded using a Jasco J-815 spectropolarimeter equipped with a temperature-controlled cell holder connected to a Peltier unit. Lectin (60 μM), prepared in 50 mM HEPES and 140 mM NaCl (pH 7.4), was preincubated in the absence or presence of either 2 mg/mL sulfatide-containing or sulfatide-free 0.4 μm liposomes. Five accumulated spectra were collected at 25 °C using a 1 mm path-length quartz cell, with a scan speed of 50 nm/min, a response time of 1 s, and a bandwidth of 1 nm.

### 2.12 Giant Unilamellar Vesicles and Confocal Microscopy

Giant unilamellar vesicles (GUVs) were generated as described by Horger et al.^25^ Briefly, an agarose solution was prepared by dissolving 4 g of agarose in 100 mL of Milli-Q water. The solution was poured onto one side of a glass slide and allowed to dry. The glass slide was then placed on a hot plate set to 38 °C. A 30 μL stock solution of lipids (3.75 mg/mL) was prepared in a chloroform–methanol mixture, containing 10% sulfatides, 30% DOPC, 30% cholesterol, 28% DOPE, and 2% DOPE-F (GUV-containing sulfatide) or 33.3% DOPC, 33.3% cholesterol, 31.3% DOPE, and 2% DOPE-F (GUV-sulfatide free). This lipid mixture was spread over the agarose layer on the heated glass slide using another glass slide. The slide remained on the hot plate for a few minutes until the solvent evaporated. To remove residual solvent, the glass slide containing the agarose and lipid film was placed in a vacuum chamber for 20 min. GUVs were then generated by placing the prepared glass slide in a Petri dish and covering it with phosphate buffer saline. The GUV-lectin suspension was prepared with a final lectin concentration of 7.5 μM. For preincubation experiments, GalNAc was added at a concentration 200 times that of lectin.

Confocal microscopy was performed using a Nikon AXR point-scanning confocal system built around an inverted Ti2 Eclipse microscope. The system was equipped with a 488 nm excitation laser and tunable GaAsP PMTs for imaging the vesicles. GUVs were imaged using a 100×/1.45 oil objective lens with a 10× scan zoom to acquire z-stacks at a step size of 0.3 μm. Representative slices were processed using ImageJ v. 1.54p, cropped to the same area size. In some cases, a gamma adjustment of 0.9 was applied to enhance fluorescence localization differences within individual vesicles. Images were adjusted for noise reduction using Adobe Photoshop 26.4.1.

### 2.13 Molecular Dynamics Simulations

We performed MDS of soybean lectin-sulfatide complexes with the polarizable AMOEBA force field.^26^ We used Poltype 2 ^27^ to compute new AMOEBA parameters for the headgroup of sulfatide. Due to its size, sulfatide was divided into three distinct fragments for parametrization. Previously established AMOEBA parameters for alkane groups were applied to the alkyl tails. When assembling the fragments, we ensured that the overall charge of the ligand remained neutral and verified the completeness of dipole and multipole moments, as well as dihedral angles, using the ANALYZE command in Tinker8 software.^28^ To validate the accuracy and stability of the derived AMOEBA parameters, we performed energy minimization using the steepest descent method, followed by a 10 ns MDS of sulfatide in a water box (65 × 65 × 65 Å^3^) at 298.15 K and 1 atm. The Nose–Hoover thermostat and barostat were employed to regulate temperature and pressure throughout the simulation. MDS were performed using Tinker9 software.^29^ The initial structure of soybean lectin was obtained from the Protein Data Bank (ID: 1G9F). Four independent systems (C1, C2, C3, and C4) were generated, each with a different placement of sulfatide. Sulfatide positioning was determined using Chimera visualization software,^30^ and periodic boundary conditions were set using PACKMOL,^31^ resulting in solvent boxes of 111 × 111 × 111 Å^3^ for each system. All systems were solvated with a pre-equilibrated water box and neutralized with nine Na^+^ ions using the GROMACS solvate algorithm.^32^ Each structure was then minimized with steepest descent, using AMOEBA, followed by MDS under constant number of particles and at 298.15 K and 1 atm. After an equilibration period of 5 ns, data were collected at 10 ns intervals for 70 ns in systems C2 and C3, whereas the systems C1 and C4 were run for 40 ns, as sulfatide in these systems gradually moved away from the protein.

### 2.14 Quantification and Statistical Analysis

Statistical analyses for all liposome co-sedimentation assays were carried out using a two-sample *t*-test, assuming equal variance. Summarized data were depicted in figures as mean ± SD from six independent experiments. Statistical significance was established as *p < 0.05.

## 3 Results

### 3.1 Isolation of Soybean Lectin and Trypsin Inhibitors in Three Steps

Lectin was successfully purified from soybean meal using galactose affinity chromatography. The bound lectin fraction was eluted and further subjected to gel filtration chromatography for additional purification (Figure 1A). To confirm the identity of the purified lectin, mass spectrometry analysis was performed on trypsin-digested fragments (Table S1), and the resulting peptide fragments matched those for soybean lectin (A0A0R0KZI2). Native gel electrophoresis confirmed that the purified lectin existed as a homotetramer, a structural state that remained unchanged upon binding to its ligand, GalNAc (Figure 1B,C). The unbound fraction from galactose affinity chromatography was enriched in KTI and BBTI. These inhibitors were separated from each other using gel filtration chromatography, resulting in distinct purified fractions (Figure 1D). This three-step procedure, comprising galactose affinity chromatography and two gel filtrations, enabled the efficient isolation of the three major antinutritional factors found in soybean meal.

**Figure 1:**
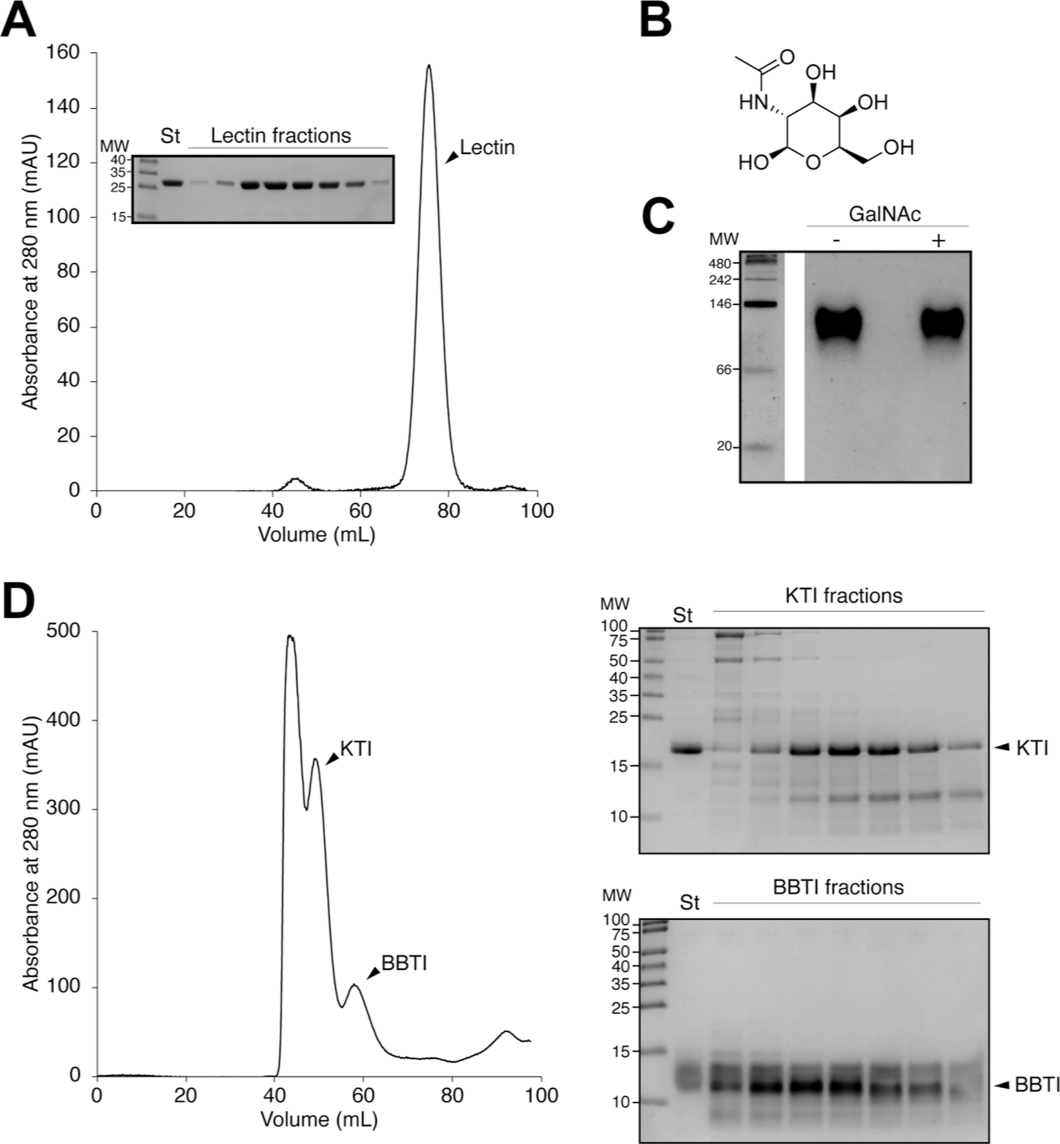
Isolation of Soybean Meal Anti-Nutritional Factors. (A) Size-exclusion chromatography (Superdex 200) profile of purified lectin. Inset: SDS-PAGE of lectin fractions from the size-exclusion chromatography. (B) Chemical structure of GalNAc. (C) Purified lectin analyzed by native gel electrophoresis in the absence and presence of GalNAc. (D) Left, size-exclusion chromatography (Superdex 30) profile of KTI and BBTI. Right, SDS-PAGE of fractions containing KTI (top) and BBTI (bottom).

### 3.2 Association of Lectin to GalNAc and Derivatives

Traditionally processed soybean meal exhibits a marked reduction in carbohydrate-binding lectin levels, ranging from 3% to 30% of those in unprocessed soybean meal, while cell–cell cross-linking activity drops to less than 10%.^33^ The interaction of soybean lectin with GalNAc promotes the inhibition of lectin-mediated agglutination of transforming cells.^34^ Whereas the specificity of soybean lectin for GalNAc has been known for some time,^10^ the thermodynamics of this association remains unknown. The ITC binding data indicate that the soybean meal lectin binds GalNAc and some of its analogs, showing variations in affinity and thermodynamic parameters (Figure 2A). GalNAc binds with a dissociation constant (*K*_D_) of 62 μM, showing a favorable enthalpic contribution (Δ*H* = −10.3 kcal/mol) and an unfavorable entropic component (−*T*Δ*S* = 4.5 kcal/mol) (Table 1). The structurally similar methyl N-acetyl deoxy α-d-galactosamine (MGalNAc) shows a similar affinity (*K*_D_ = 73 μM) (Figure 2B and Table 1), suggesting that methylation at the anomeric position does not markedly alter binding. However, 2-nitro-phenyl β-d-galactopyranoside (NPGP), a β-linked derivative, and lacto-N-tetraose (L-NT) bind with ∼10-fold weaker affinities, respectively (Figure 2C,D), highlighting the preference of soybean lectin for a less bulky substitution at the anomeric carbon of GalNAc. No binding was observed for 4-nitro-phenyl α-d-mannopyranoside (NPMP), octyl α-D mannopyranoside (OMP), 2-chloro-4-nitromannopyranoside (CNMP), or 6-sialyl lactose (6-SL) (Figure S1A–D), reinforcing that, unlike animal lectins,^35^ the specificity of soybean lectin is for GalNAc over mannose or sialylated ligands.

**Figure 2:**
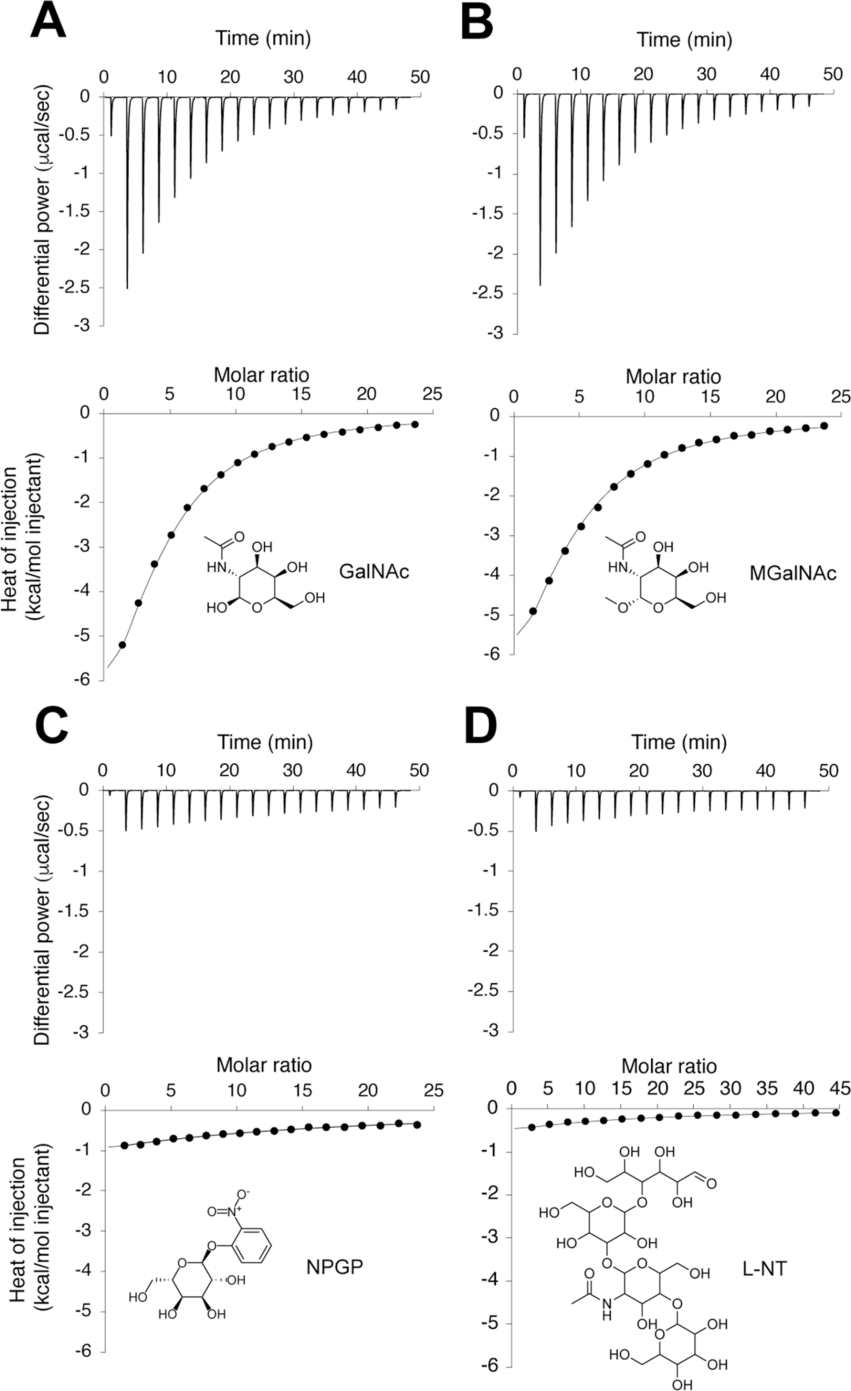
Binding of lectin to GalNAc and its analogs. ITC thermograms of GalNAc (A), MGalNAc (B), NPGP (C), L-NT (D) for binding to soybean lectin. In all cases, the top panel represents the ITC raw binding data for protein interactions, whereas the bottom is the integrated and normalized data fit with a one set of sites binding model, with N fixed to 4.

**Table 1:** Thermodynamic Parameters for the Binding of Soybean Lectin to GalNAc, Structural Analogs, and the Modulatory Role of sulfatide^a^

ligands^b^	*K*_D_ (μM)	Δ*H* (kcal/mol)	Δ*G* (kcal/mol)	–*T*Δ*S* (kcal/mol)
GalNAc	61.6 ± 0.1	–10.3 ± 0.1	–5.8 ± 0	4.5 ± 0.1
MGalNAc	73.1 ± 0.1	–10.7 ± 0.1	–5.7 ± 0	5.0 ± 0.1
NPGP	789.5 ± 0.7	–9.8 ± 0.4	–4.3 ± 0	5.5 ± 0.3
L-NT	770 ± 10	–5.1 ± 0.5	–4.2 ± 0	0.9 ± 0.5
NPMP	NB^c^			
OMP	NB			
CNMP	NB			
6-SL	NB			
GalNAc + sulfatide liposomes	78.9 ± 5.4	–9.9 ± 0.2	–5.6 ± 0	4.3 ± 0.2
GalNAc + sulfatide-free liposomes	80.8 ± 1.5	–9.9 ± 0.2	–5.6 ± 0	4.3 ± 0.2

a Values represent the mean of at least two independent experiments. Errors are displayed as standard deviation values.

b GalNAc, *N*-acetylgalactosamine; MGalNAc, methyl *N*-acetyl deoxy α-d-galactosamine; NPGP, 2-nitrophenyl β-d-galactopyranoside; L-T, lacto-*N*-neotetraose; NPMP, 4-nitrophenyl α-d-mannopyranoside; OMP, octyl α-D mannopyranoside; CNMP, 2-chloro-4-nitromannopyranoside; 6-SL, 6-sialyl lactose.

c NB, no binding detected (for *K*_D_ over 1–2 mM).

We also investigated whether TI activity or concentration correlates with lectin activity in soybean meal variants. To do this, we used four soybean lines with distinct KTI and BBTI levels.^24^ Our findings indicate that the affinity of lectin binding to GalNAc remain unchanged regardless of TI concentration or affinity for trypsin (Table S2). These results suggest that the antinutritional activity of lectin in soybeans is independent of that exhibited by TIs.

### 3.3 Binding of Lectin to the Glycosphingolipid Sulfatide

Lectins are generally known to associate with membranes by interacting with glycosylated receptors and glycolipids. To investigate whether they specifically interact with sulfatide-containing membranes, we prepared sulfatide liposomes with varying curvatures. Liposomes of different sizes were generated by stepwise extrusion through filter membranes with pore sizes of 0.4, 0.2, 0.1, and 0.05 μm. DLS analysis revealed that liposome sizes closely matched the pore sizes for 0.4 and 0.2 μm filters but were larger than expected for the 0.05 and 0.1 μm filters (Figure 3A). Liposomal size distributions often deviate slightly from the expected values. Specifically, liposomes extruded through membranes with pore sizes of 0.2 μm and above typically yield vesicles smaller than the pore size, whereas membranes with pore sizes below 0.2 μm tend to produce liposomes larger than expected.^36^ This behavior is attributed to the elastic deformation of liposomes from spherical to ellipsoidal shapes,^37^ which facilitates their passage through membrane pores.^38^ Among the tested liposomes, lectin showed the strongest binding to 0.4 μm pore-size liposomes (Figure 3B). To quantitatively evaluate sulfatide binding, we immobilized 0.4 μm sulfatide liposomes onto a sensor chip and measured the kinetic properties of lectin binding (Figure 3C). Global fitting of the binding curves using a two-reaction binding model yielded a *K*_D_ of 264 ± 62 nM (χ^2^ = 2.1). The maximum binding response was ∼75 RU, close to the theoretical maximum of 128 RU predicted by the model, which assumes initial protein–ligand binding followed by a conformational change. To analyze the potential sulfatide-dependent conformational changes of lectin, we collected near-UV circular dichroism spectra. Lectin displayed a positive ellipticity signal due to its aromatic residues found in unique asymmetric environments (Figure 3D). Unlike sulfatide-free liposomes, binding of lectin to sulfatide-containing liposomes induced minor peak shifts in the 270–290 nm region, primarily associated with local conformational changes in protein regions containing tyrosine and tryptophan residues. To determine whether GalNAc and sulfatide compete for soybean lectin binding, we performed preincubation experiments with one of the ligands. Preincubating lectin with an excess of GalNAc did not affect its affinity for sulfatide liposomes, nor did preincubation with sulfatide liposomes alter lectin’s binding to GalNAc (Figure S2 and Table S3). Thus, the local conformational changes induced by sulfatide binding did not interfere with the ability of lectin to bind GalNAc.

**Figure 3:**
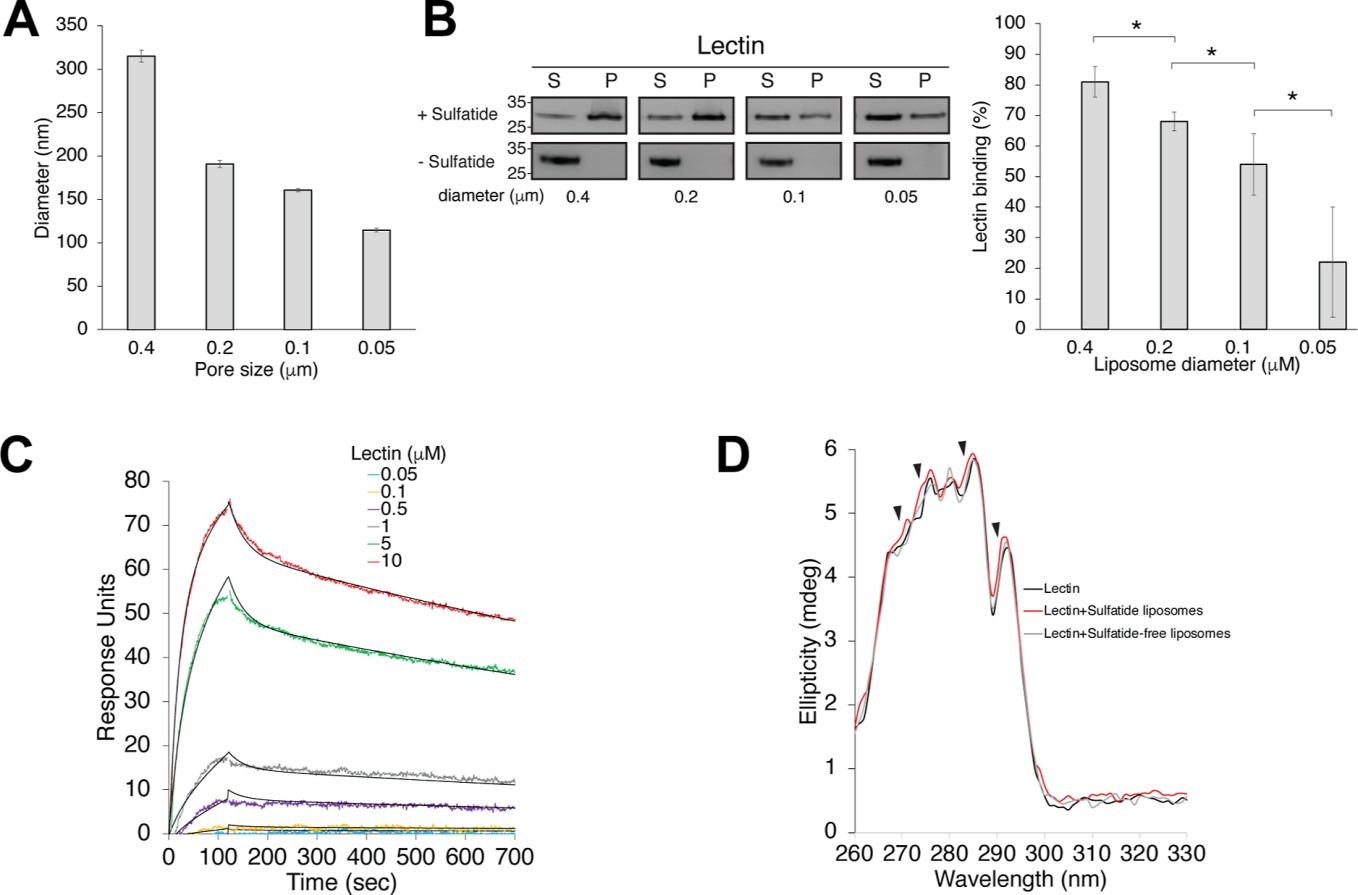
Soybean lectin binds sulfatide in a membrane-curvature-dependent manner. (A) Analysis of the average diameters of the indicated sulfatide-containing liposome sizes, as evaluated by DLS. (B) Effect of the liposome size on the binding of lectin to sulfatide-containing liposomes. Left, analysis of the liposome co-sedimentation assay for lectin with sulfatide-containing and sulfatide-free liposomes S, supernatant; P, pellet. Right, quantification of the intensity of the lectin bands obtained by densitometry. (C) SPR sensorgrams for the interaction of lectin with sulfatide liposomes. (D) Near-UV CD spectra of lectin in the absence and presence of either sulfatide-containing or sulfatide-free liposomes.

### 3.4 Sulfatide-Dependent Activity of Lectin

Fluorescently labeled giant unilamellar vesicles (GUVs) enriched with sulfatide (∼0.5–1 μm in diameter) were incubated with soybean lectin, and their interactions were monitored using confocal microscopy. As shown in Figure 4A,B, sulfatide-containing GUVs underwent progressive agglutination upon soybean lectin addition, accompanied by membrane shape changes at contact sites. Fluorescence intensity at these contact sites increased markedly, likely due to bilayer proximity rather than lateral DOPE redistribution. Soybean lectin-induced cross-linking of sulfatide-containing GUVs closely resembled bacterium LecA-mediated agglutination of sphingolipid-enriched GUVs,^39^ suggesting a conserved agglutination mechanism despite differences in amino acid composition. No aggregation occurred in the absence of lectin (Figure 4A). The addition of excess GalNAc inhibited soybean lectin-mediated cross-linking, suggesting that the lectin-mediated membrane cross-linking activity is located near or at the GalNAc-binding site (Figure 4C). No evident lectin-mediated GUV cross-linking was observed when sulfatide was omitted (Figure 4D,E). Taken together, these findings demonstrate that sulfatide promotes soybean lectin-mediated membrane cross-linking, a process that can be blocked by GalNAc.

**Figure 4:**
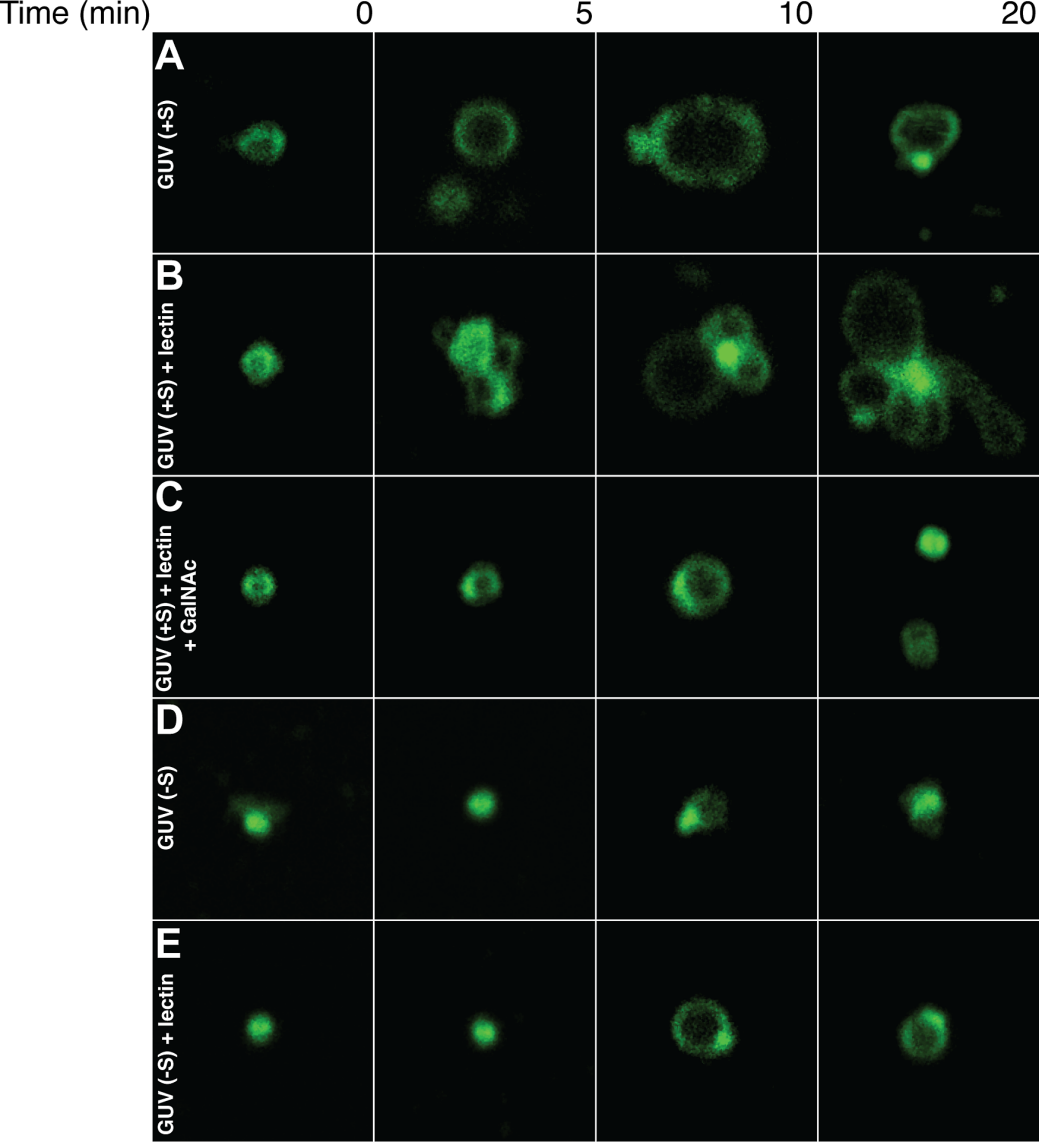
Time-dependent interaction of lectin with sulfatide-enriched GUVs. Representative confocal images showing time-dependent GUVs under the following conditions: (A) GUV [+S]: Sulfatide-enriched GUVs; (B) GUV [+S] + lectin: Sulfatide-enriched GUVs in the presence of soybean lectin; (C) GUV [+S] + lectin + GalNAc: Sulfatide-enriched GUVs in the presence of lectin and an excess of GalNac; (D) GUV [-S]: GUVs lacking sulfatide; and (E) GUV [-S] + lectin: GUVs lacking sulfatide in the presence of lectin.

### 3.5 Identification of the Sulfatide Binding Site in Lectin

To identify the sulfatide-binding site in soybean lectin, we performed MDS using the tertiary structure of the protein deposited in PDB (ID#1G9F). At *t* = 0, sulfatide was placed at four distinct positions around the protein (C1, C2, C3, and C4; Figure S3). In the C1 and C4 simulations, sulfatide detached from lectin within the first 40 ns (Figures S4A,C and S5). In contrast, sulfatide remained in closer proximity to lectin in the C2 and C3 simulations (Figures 5A, S4B and S5). To assess these interactions, we measured the frequency with which the distance between selected sulfatide atoms and lectin heavy atoms fell below 6.5 Å. Our data reveals that in C2, the glycosphingolipid explores a broader region around lectin (Figure S4B), engaging in only a few specific interactions with the headgroup, primarily within CBD (Figure 5B). However, since GalNAc did not compete with sulfatide for lectin binding (Figure S2), it is unlikely that C2 represents the mechanism for sulfatide binding for soybean lectin. In contrast, the C3 simulation suggests a more localized binding site (Figures 5A and S5), where sulfatide employs its sulfate headgroup for protein targeting. Specifically, residues I149, T151, T152, W154, Y181, and R185 interact with the lipid headgroup in C3 (Figure 5C). Also, several lectin residues interact with the alkyl tails of sulfatide, including K10, D155, L156, N158, L179, V180, T245, F247, and L249. Interestingly, many of these sulfatide-interacting residues align with the canonical sphingolipid-binding domains reported for Disabled-2, α-synuclein, and mesencephalic astrocyte-derived neurotrophic factor (MANF).^19^ In particular, lectin residues Y181 and R185 (Figure 5D) correspond to highly conserved residues that are critical for sulfatide interactions.^19^

**Figure 5:**
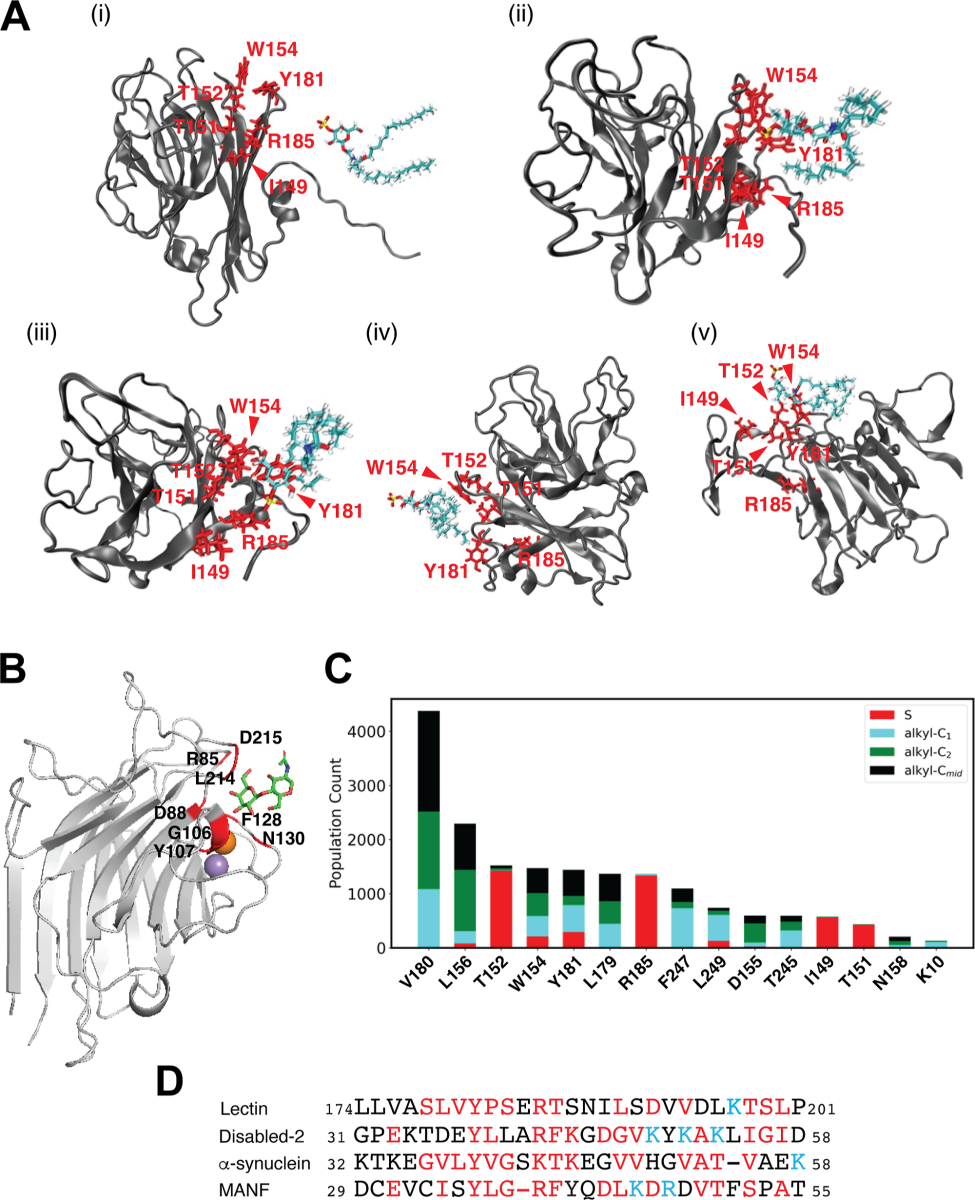
Identification of the sulfatide-binding site in soybean lectin. (A) Snapshots from MDS of soybean lectin with sulfatide at 0 (i), 20 (ii), 40 (iii), 60 (iv), and 80 ns (v). Sulfatide-interacting residues are highlighted in red. Sulfatide is in a stick representation. (B) Structural model of soybean lectin bound to GalNAc. Residues forming the GalNAc binding site within CBD are labeled. GalNAc is represented as a stick model with Ca^2+^ in orange and Mn^2+^ in purple. (C) Histogram showing the frequency of lectin residues interacting with different regions of sulfatide, based on the distance between any heavy atom of the protein and selected sulfatide atoms. (D) Protein sequence alignment of the sphingolipid-binding domain in the indicated proteins, with conserved residues highlighted in red. Sequence alignment was generated using COBALT with default advanced parameters, except for gap penalties set to −3 (opening) and −4 (extension), and end-gap penalties set to −3 (opening) and −3 (extension).

## 4 Discussion

We developed a straightforward three-step chromatographic procedure to isolate the three major antinutritional factors in soybean meal—lectin, KTI, and BBTI. Our lectin purification procedure involved four steps and yielded 1.09 mg per gram of soybean meal. Whereas this yield is comparable to those reported for other soybean lectin purification methods,^40^ our approach, which also included the concurrent purification of lectin, KTI, and BBTI, was markedly more time efficient, completing the entire process in under 24 h. The purified lectin was tetrameric, consistent with its known structure in soybean seeds,^41^ and remained in this state in the presence of its ligand, GalNAc. Although the binding preference of soybean lectin for GalNAc has been previously established,^10^ we report, to our knowledge, the first direct binding of purified soybean lectin to this ligand. ITC revealed that lectin bound GalNAc exothermically, with a *K*_D_ of 62 μM. The observed monotonic decrease in heat release with increasing GalNAc concentration suggests that soybean lectin displays a single type of binding site and exhibits no allosteric interactions among the four sites on the tetramer. The simultaneous presence of lectin and TIs in soybean meal lines did not appear to involve crosstalk, as changes in TIs levels did not affect lectin binding activity for GalNAc.

Soybean lectin contains a single CBD in each monomer that recognizes sugar moieties in coordination with Ca^2+^ and Mn^2+^ ions. This CBD is highly conserved, with 50% of its residues invariant among legume lectins.^42^ Four of these residues -Asp, Gly, Asn, and Phe (or Tyr)- determine sugar specificity^9^ (Figure 5B). In soybean lectin, D88 forms hydrogen bonds with the C3–OH and C4–OH hydroxyl groups of GalNAc, whereas the carbonyl group of its N-acetamide moiety interacts with the NH backbones of G106 and N130 ^9^. In addition, the C4–OH group forms a hydrogen bond with the backbone NH of L214, and the C6–OH hydroxyl group interacts with R85 and D215. The remaining key lectin residue, F128, stabilizes the sugar by stacking against its lower face, engaging C1–H, C3–H, and C5–H groups. Further support comes from the N-acetamide group’s interaction with Y107, which may explain why lectin displays a higher affinity for GalNAc than galactose.^9^ In addition, the methylation of GalNAc (MGalNAc) likely affects only the accessibility of the C1–H group, which does not play a major role in GalNAc’s function. This explains why MGalNAc exhibits a similar affinity for lectin compared to GalNAc (Table 1), closely matching the affinity reported for a lectin from Pseudomonas aeruginosa.^43^

Very few studies have reported structural details of lectin interactions with GalNAc derivatives. Unlike soybean lectin, the plant lectin concanavalin A is specific to α-d-mannose and α-d-glucose,^44^ in part due to differences in oligomeric stability and glycosylation patterns. The presence of two tyrosine residues in the CBD of concanavalin A, which are absent in soybean lectin, favors hydrophobic interactions with the paranitrophenyl group of NPMP.^45^ Thus, this structural difference may explain why soybean lectin does not recognize NPMP. Both human galectin-4 and galectin-8 have been shown to bind L-NT with higher affinity than soybean lectin.^46^ L-NT is conjugated to the blood group antigen and the natural killer antigen found on hematopoietic cells. Galectin-4 and -8 contain two CBDs arranged in tandem, connected by a flexible linker of variable length,^47^ which may explain their ability to accommodate larger GalNAc derivatives in their binding pockets.

Galectins play a role in cellular organization by forming cross-linked complexes, known as galectin lattices, through their binding to glycosylated receptors on the cell surface. This lattice formation influences the diffusion, compartmentalization, and endocytosis of glycoproteins and glycolipids, ultimately affecting cellular signaling and immune responses.^48^ One proposed mechanism, known as the glycolipid-lectin hypothesis, suggests that galectins facilitate the co-clustering of glycolipids and glycoproteins into membrane nanodomains. This organization enables endocytosis via clathrin-independent pathways, a process essential for cellular uptake and signal transduction.^13^ Interestingly, galectins employ self-association to enhance their biological function. For example, although galectin-3 is monomeric, it can oligomerize through hydrophobic interactions via its intrinsically disordered N-terminal domain.^49^ This level of organization allows galectin-3 to commit for dynamic interactions and phase separation, thereby strengthening its binding capabilities.

In this study, we show that soybean lectin interacts with the glycosphingolipid sulfatide with high affinity. Sulfatide is abundant in intestinal epithelial membranes and contributes to membrane stability, signaling, and immune regulation.^19^ Thus, lectin-sulfatide interactions could disrupt lipid raft organization, nutrient transport, and mucosal inflammation,^50^ such as the case for the observed morphology alteration of the intestine and barrier function in rats.^51^ A limited number of studies have examined the cellular consequences of lectin association with membrane glycolipid sulfatide. The lectin domain of brevican, a nervous system-specific proteoglycan, binds membrane sulfatide and sulfoglucuronylglycolipids in a Ca^2+^-dependent manner, facilitating cell-substrate interactions essential for migration and adhesion processes.^52^

Here, we demonstrate that soybean lectin, a highly stable tetramer, binds GalNAc and sulfatide liposomes in a noncooperative manner, suggesting that these ligands target distinct, nonoverlapping binding sites. Furthermore, the lack of cooperativity between ligands is reinforced by the finding that sulfatide does not induce major conformational changes in soybean lectin (Figure 3D), minimizing the likelihood of affecting GalNAc binding. Early work by Read and colleagues demonstrated that jack bean Concanavalin A binds to a glycolipid isolated from biological membranes.^53^ Unlike soybean lectin, the interaction was inhibited by α-methyl mannose, indicating that the binding involves mannose-containing glycan structures on the glycolipid. Evidence supporting the existence of independent carbohydrate and glycolipid binding sites in lectins is found in C-type lectins, which accommodate different ligands through secondary binding sites located away from their primary CBD. These secondary sites contribute to the functional diversity of C-type lectins, enabling them to interact with a variety of ligands, including glycolipids and proteins.^54^ The high affinity of soybean lectin for sulfatide, as determined by SPR measurements, suggests that this lipid serves as a platform for recruiting the protein to the host membrane, thereby facilitating its interaction with GalNAc-conjugated receptors.

Soybean lectin was observed to cluster sulfatide-containing GUVs (Figure 4). Other lectins have also been implicated in membrane interactions and remodeling. For instance, Helix pomatia lectin can cross-link membranes, with adhesion strength modulated by membrane tension and curvature.^55^ The β-subunit of the Shigella dysenteriae Shiga toxin induces membrane bending upon binding to the glycosphingolipid globotriaosylceramide.^56^ In contrast to soybean lectin and Shiga toxin, the monomeric animal galectin-3 requires glycosylated proteins for membrane recruitment, leading to tubular membrane invaginations.^14^ Preincubation with an excess of GalNAc reduced lectin-mediated cross-linking of sulfatide-containing GUVs. However, our observations indicate that sulfatide and GalNAc do not compete for binding to lectin (Figure S2). Thus, although the sulfatide binding site remains accessible, GalNAc may sterically interfere with the lectin’s ability to cross-link sulfatide-containing membranes. This suggests a mechanism similar to noncompetitive inhibition, where function is impaired through interactions that do not involve the binding site directly.

Our MDS suggest that sulfatide interacts with soybean lectin at a specific binding site, primarily observed in the C3 simulation. Whereas sulfatide explored a broader region in C2, its lack of competition with GalNAc suggests that C2 does not represent the primary binding site. Instead, the localized interactions in C3, particularly within a predominantly positively charged region distinct from the GalNAc binding site (Figure S6), suggest a more likely sulfatide-binding site. These findings align with known sphingolipid-binding domains in other proteins,^19^ reinforcing the relevance of conserved residues in sulfatide recognition. Future studies using site-directed mutagenesis followed by sulfatide-binding assays could validate the functional significance of these residues.

In summary, this report reveals a dual recognition mechanism for soybean lectin, demonstrating selective GalNAc binding and a novel, high-affinity interaction with sulfatide-containing membranes. These findings offer new molecular insights into lectin-mediated cell adhesion and signaling, with potential implications for understanding its antinutritional effects in soybean-based diets for both animals and humans.

## Supplementary Material



## Notes

ITC and SPR data showing the lack of competition between GalNAc and sulfatide for lectin binding, MDS set up for sulfatide binding to lectin; additional MDS results when sulfatide was positioned in distinct places around lectin, electrostatic surface analysis of the GalNAc and sulfatide binding sites on lectin, identification of lectin peptides by mass spectrometry, analysis showing no correlation between GalNAc-binding activity and TI concentration across different soybean meal lines, comparison of lectin binding affinity to sulfatide-enriched liposomes in the absence and presence of GalNAc using SPR (PDF)
