# Supplementary material for: Soybean Lectin Cross-Links Membranes by Binding Sulfatide in a Curvature-Dependent Manner
Source: J Agric Food Chem. 2025 May 24;73(22):14020–31. doi: 10.1021/acs.jafc.5c04336 (PMC12147205; doi:10.1021/acs.jafc.5c04336)
Supplement: Supplementary file 1 [file jf5c04336_si_001.pdf]

SUPPORTING INFORMATION

Soybean lectin crosslinks membranes by binding sulfatide in a curvature-dependent manner

Ayoyinka O. Okedigba, Emery L. Ng, Mawuli Deegbey, M. Luciana Rosso, William Ngo, Ruoshi Xiao, Haibo Huang, Bo Zhang, Valerie Vaissier Welborn, Daniel G. S. Capelluto*

* Corresponding author: Protein Signaling Domains Laboratory, Department of Biological Sciences, Fralin Life Sciences Institute, Virginia Tech, Blacksburg, VA 24061, United States.

E-mail address: capellut@vt.edu (D. G. S. Capelluto).

The file contains the following:

Figures S1-S6

Tables S1-S3

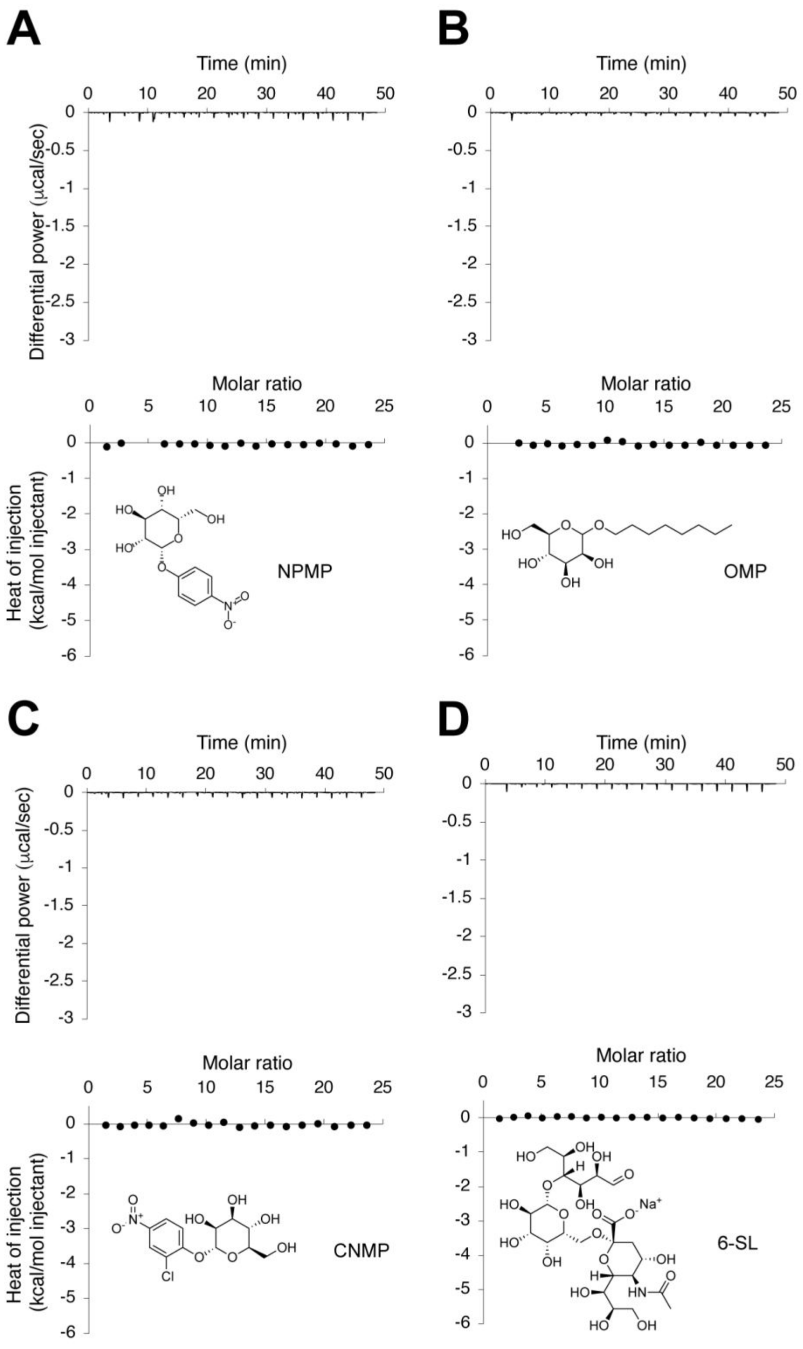

Figure S1. Soybean lectin does not bind selected GalNAc derivatives. ITC thermograms showing no detectable binding between soybean lectin and four GalNAc-related compounds: NPMP (**A**), OMP (**B**), CNMP (**C**), and 6-SL (**D**). For each compound, the upper panels show raw differential power over time, while the lower panels display the integrated heat per injection plotted against molar ratio. Data were analyzed using a one-set-of-sites binding model with the stoichiometry (N) fixed at 4.

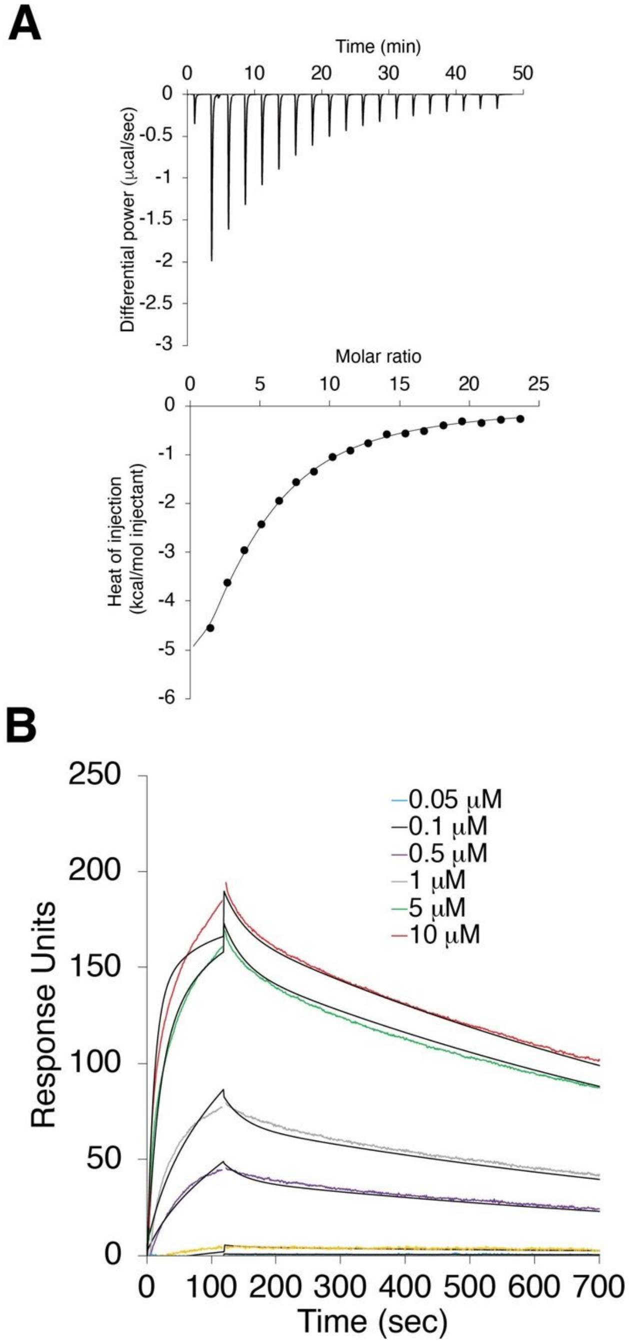

Figure S2. GalNAc and sulfatide do not compete for binding to soybean lectin. (**A**) ITC analysis of lectin binding to GalNAc in the presence of excess of sulfatide-containing liposomes. (**B)** SPR analysis of lectin binding to sulfatide-containing liposomes in the presence of excess of GalNAc.

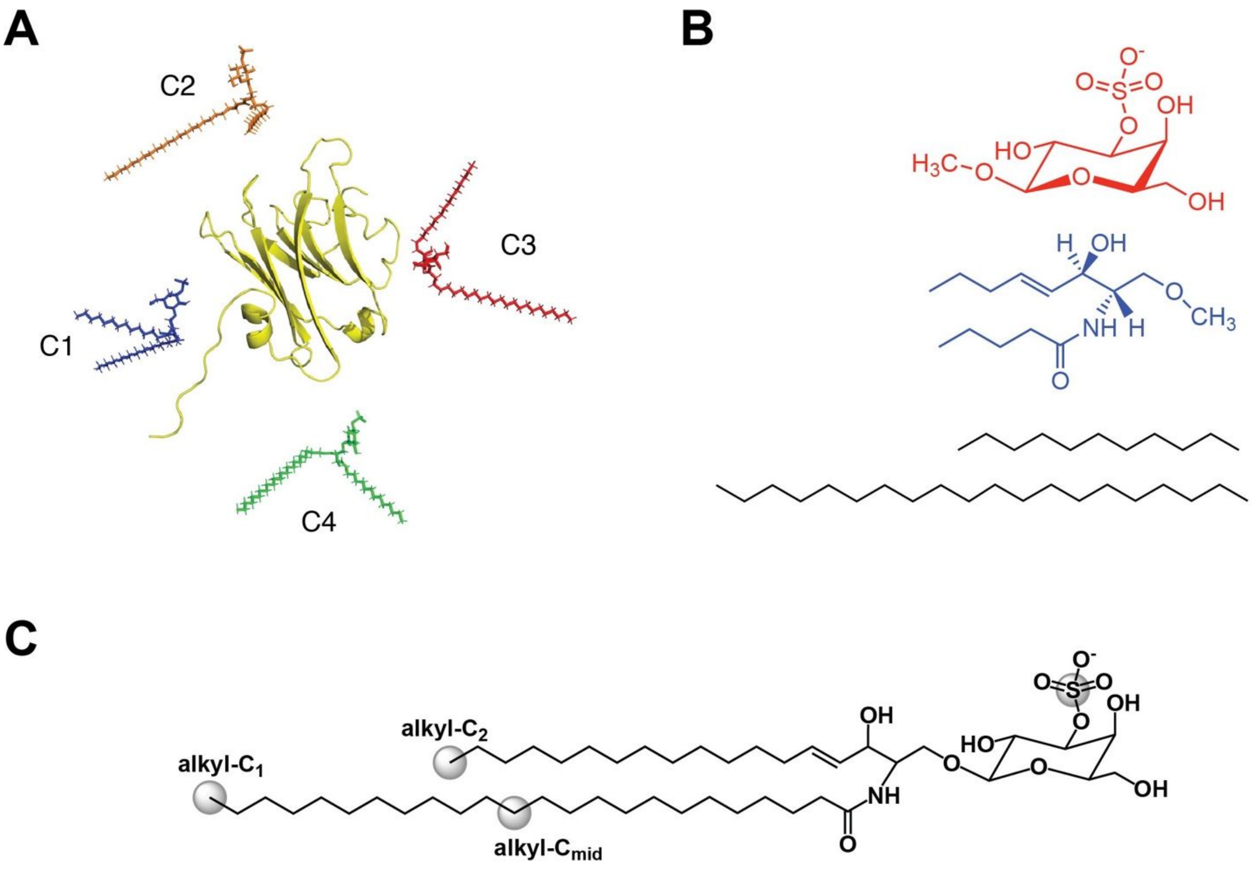

Figure S3. MDS set up for lectin binding to sulfatide. (**A**) Cartoon representation of soybean lectin (yellow) and initial sulfatide configurations: C1 (red), C2 (orange), C3 (blue), and C4 (green). (**B**) Sulfatide fragments used for AMOEBA parameterization (**C**) Chemical structure of sulfatide, with highlighted atom groups selected for binding analysis.

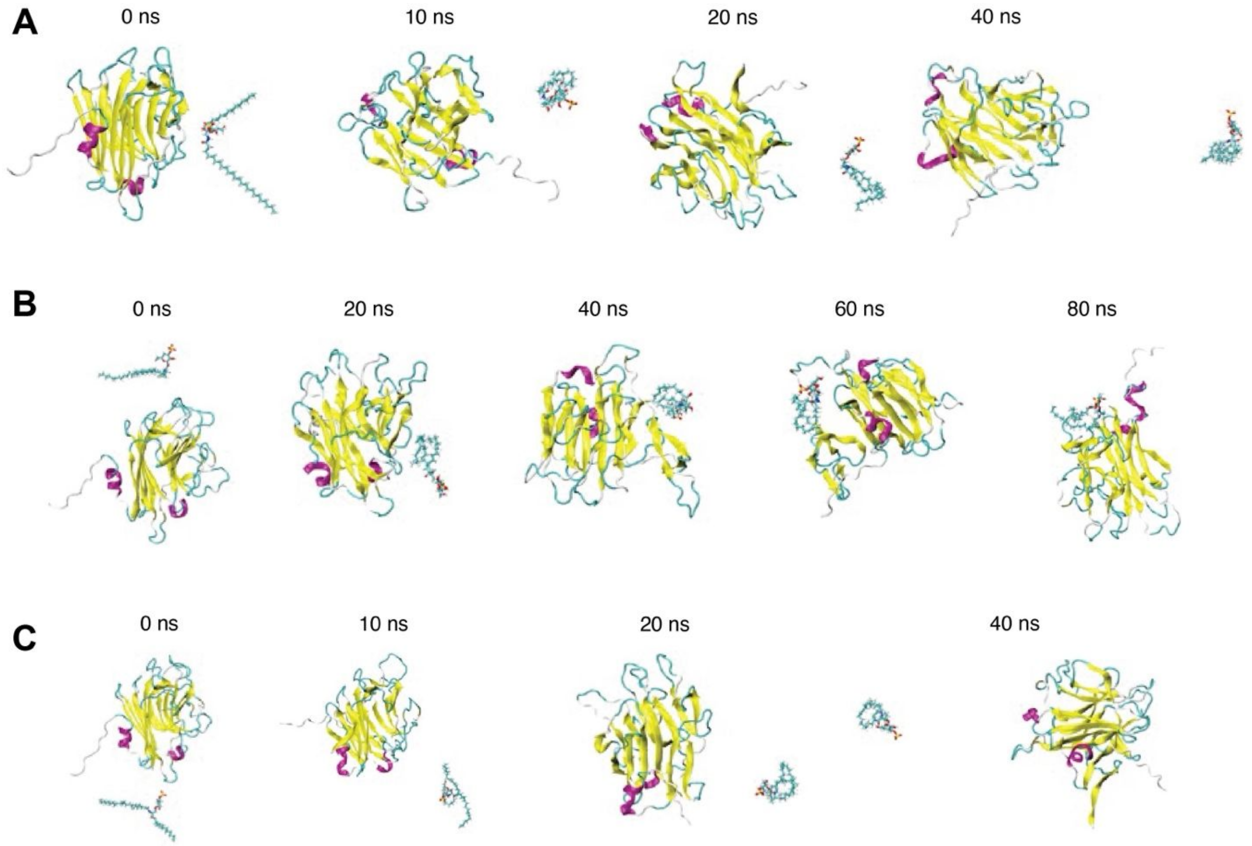

Figure S4. Additional MDS performed in this study. Snapshots of soybean lectin in the presence of sulfatide at the indicated time points for simulations C1 (**A**), C2 (**B**), and C4 (**C**), shown as cartoon representations.

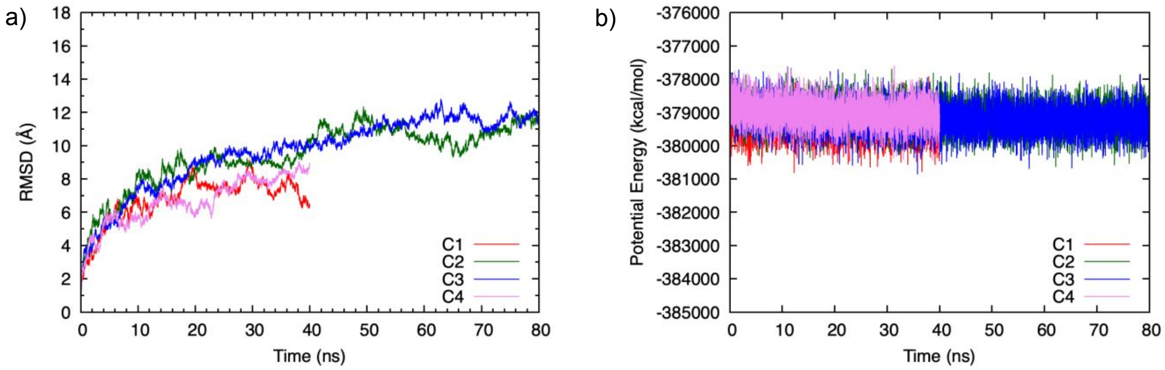

Figure S5. (**A**) Root-mean-square deviation (RMSD) profiles over time and (**B**) Energy convergence plots demonstrating the equilibration behavior for each simulated systems.

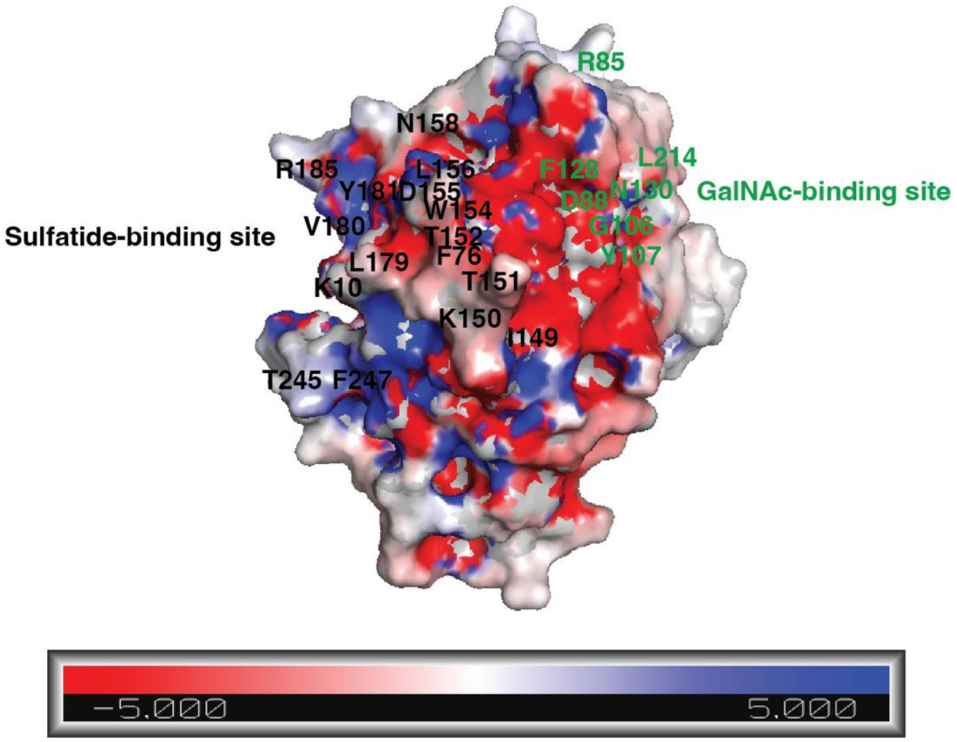

Figure S6. Potential binding sites for sulfatide and GalNAc in soybean lectin. The surface potential was generated by applying APBS electrostatics in PyMol. Residues involved in sulfatide binding are labeled in black, whereas those engaged in GalNAc binding are marked in green.

Table S1. Identification of soybean lectin. MS/MS results obtained on peptides generated by trypsin proteolysis of lectin.

Peptide	Lectin	Sequence
1	15-31	QPNMILQGDAIVTSSGK
2	37-50	VDENGTPKPSSLGR
3	151-160	TTSWDLANNK
4	186-197	TSNILSDVVDLK
5	198-205	TSLPEWVR

Table S2. Thermodynamic parameters for NAG-soybean meal interactions and comparison with porcine trypsin-soybean meal TIs interactions. Values represent the mean of at least two independent experiments. Errors are displayed as standard deviation values. K_D, dissociation constant.

Soybean Meal Line	TIs	TI concentration (mg/g meal)	K_D (μM) TI to trypsin	K_D (μM) Lectin to GalNAc
Glenn	BBTI	4.6	0.1 ± 0	66.5 ± 0.4
	KTI	8.2	1.1 ± 0.4	
17Ti-0204	BBTI	5.6	0.1 ± 0	68 ± 2
	KTI	0.6	9 ± 2	
17Ti-0242	BBTI	4.0	0.1 ± 0.1	66 ± 3
	KTI	0.5	2.4 ± 0.3	
17Ti-0332	BBTI	4.8	0.2 ± 0	71.4 ± 0
	KTI	6.5	1.2 ± 0.1	
17Ti-0534	BBTI	4.8	0.1 ± 0	69.2 ± 0.5
	KTI	10.3	2.4 ± 0.1	

Table S3. Kinetic analyses for the interaction of lectin to sulfatide liposomes in the absence and presence of 1 mM of GalNAc.

GalNAc	K_D (nM)	Chi^2	Theoretical R_{max}
-	264±62	2.2	128
+	226±1	4.5	164
